# What if They Don’t Have Tuberculosis? The Consequences and Trade-offs Involved in False-positive Diagnoses of Tuberculosis

**DOI:** 10.1093/cid/ciy544

**Published:** 2018-07-05

**Authors:** Rein M G J Houben, Marek Lalli, Katharina Kranzer, Nick A Menzies, Samuel G Schumacher, David W Dowdy

**Affiliations:** 1 Tuberculosis Modelling Group, Tuberculosis Centre and Centre for Mathematical Modelling of Infectious Diseases; 2 Infectious Disease Epidemiology Department, Faculty of Epidemiology and Public Health, London School of Hygiene and Tropical Medicine, United Kingdom; 3 Research Centre Borstel, National and Supranational Reference Laboratory, Germany; 4 Department of Global Health and Population, Harvard T. H. Chan School of Public Health, Boston, Massachusetts; 5 Foundation for Innovative New Diagnostics, Geneva, Switzerland; 6 Department of Epidemiology, Johns Hopkins Bloomberg School of Public Health, Baltimore, Maryland

**Keywords:** tuberculosis, false-positive diagnosis, trade-off

## Abstract

To find the millions of missed tuberculosis (TB) cases, national TB programs are under pressure to expand TB disease screening and to target populations with lower disease prevalence. Together with imperfect performance and application of existing diagnostic tools, including empirical diagnosis, broader screening risks placing individuals without TB on prolonged treatment. These false-positive diagnoses have profound consequences for TB patients and prevention efforts, yet are usually overlooked in policy decision making. In this article we describe the pathways to a false-positive TB diagnosis, including trade-offs involved in the development and application of diagnostic algorithms. We then consider the wide range of potential consequences for individuals, households, health systems, and reliability of surveillance data. Finally, we suggest practical steps that the TB community can take to reduce the frequency and potential harms of false-positive TB diagnosis and to more explicitly assess the trade-offs involved in the screening and diagnostic process.

## Teaser

False-positive diagnoses are frequent in tuberculosis and have profound consequences, yet are usually overlooked in policy decision making. We describe pathways and trade-offs involved, and their consequences for individuals, households, and health systems. We then discuss steps to mitigate their impact.

To achieve ambitious targets to reduce incidence and deaths due to tuberculosis (TB), global and national policies emphasize the need to diagnose and treat a larger fraction of the 10.4 million individuals who develop TB disease each year [1, 2]. For this reason, national tuberculosis programs are under pressure to expand access to TB screening and diagnosis [3]. Similarly, the need for more sensitive diagnostic tools to detect patients earlier in the course of their disease and care-seeking process has been highlighted, but increased sensitivity may come at the cost of reduced specificity [4, 5].

Current TB policy discussions, program targets, and indicators usually do not consider the risk of false-positive TB diagnoses [2, 6, 7], despite recognition of the issue in World Health Organization (WHO) recommendations of systematic screening programs [8].

In this article we describe how false-positive diagnoses are part of TB clinical practice, along with their consequences for individuals, households, health systems, and surveillance data. Our aim is to enable comprehensive discussions on the trade-offs involved for expanded TB screening programs. We then propose concrete actions that can be undertaken to mitigate the negative effects of false-positive TB diagnosis.

## Definition of False-positive TB Diagnosis

We define a false-positive TB diagnosis as one where an individual, who does not have active TB disease, incorrectly receives a diagnosis of TB disease. In this vein, we use the term “false-positive” (a term widely used in clinical epidemiology) to suggest the absence of active TB disease, not the absence of symptoms or the absence of other non-TB illness (eg, bacterial pneumonia, obstructive lung disease). Importantly also, we use this term also to apply only to the diagnostic process itself, not the individual undergoing diagnosis, as the latter may be stigmatizing [9, 10].

## Path Toward False-positive TB Diagnosis

**Figure 1.**
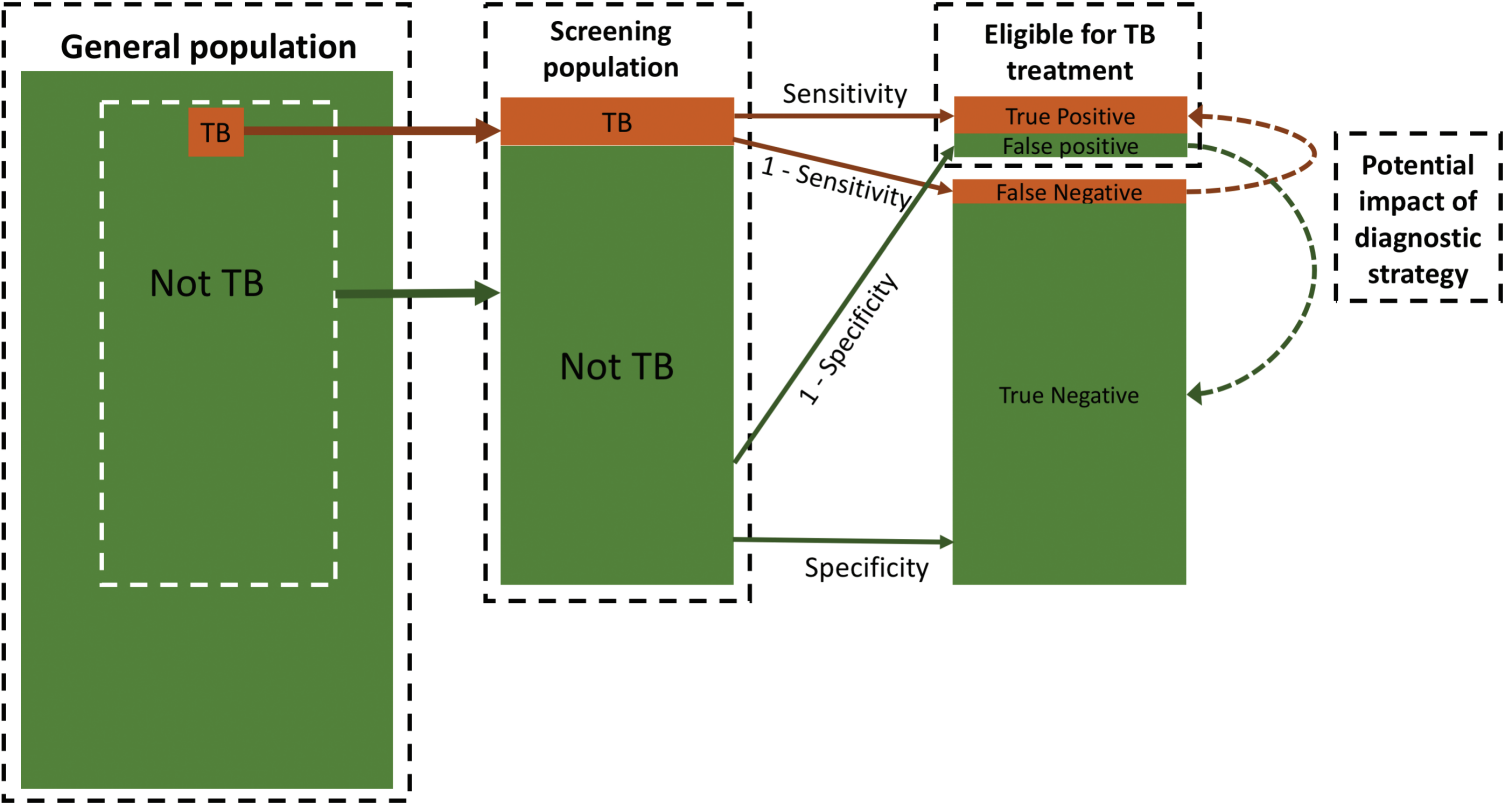
Screening and diagnostic pathway for tuberculosis (TB). From a general population, a screening population is formed from individuals with (orange) and without (green) TB. The diagnostic algorithm is applied to the screening population, categorizing individuals into those recommended for TB treatment (following a true-positive or false-positive diagnosis) or not. The contribution of false-positive TB diagnoses is mostly driven by the prevalence of TB in the screening population and the specificity of the diagnostic algorithm (see Table 1). The dashed arrows on the right highlight the 2 processes that new screening or diagnostic strategies aim to achieve (orange = convert false-negative diagnoses into true-positive diagnoses; green = convert false-positive diagnoses into true-negative diagnoses).

Figure 1 outlines the screening and diagnostic pathway for TB. A screening population is composed of individuals with and without TB. Some individuals without TB may have an underlying illness that presents with similar symptoms to those of TB. A diagnostic algorithm, with imperfect sensitivity and specificity, is then applied to the screening population. All those diagnosed with TB (correctly or not) are eligible to start treatment and should be notified as part of national and global surveillance.

The proportion and the underlying reasons for false-positive TB test results vary, and are highly setting-dependent as local guidelines and policy will dictate different diagnostic algorithms in different populations. Differences in background prevalence of TB or comorbidity patterns (eg, human immunodeficiency virus [HIV], silicosis) as well as human performance and laboratory capacity can influence test performance and result interpretation. A key example is empirical (or “clincal”) diagnosis, where TB is diagnosed (and treatment is started) in the absence of a recorded positive bacteriological test. Empirical diagnosis accounted for 43% of all cases reported to WHO globally in 2017 [7]. Decisions to treat empirically are based on a mix of symptomatic presentation, comorbidities, chest radiography and other ancillary tests, nonresponse to other therapeutic maneuvers (eg, trials of broad-spectrum antibiotics), and individual clinician assessment—all of which vary from one population (and one provider) to the next. Empirical diagnosis is a critical part of the diagnostic arsenal, especially in low-resource settings where health workers may see a high number of TB patients to inform their clinical judgement. However, the limited data available suggest that the sensitivity and specificity of empirical diagnosis are both highly variable and suboptimal. A multicountry diagnostics trial found that sensitivity ranged from 16% to 44.4% and the specificity ranged from 86.9% to 95.3% across study sites, and was significantly influenced by coverage of chest radiography [11].

In high-income settings, where greater resources are available, initial screening and diagnostic approach are strongly tilted toward increasing specificity, thus decreasing the risk of a false-positive diagnosis despite the low prior of TB in the screening population.

By contrast, TB diagnosis in a rural health center in a resource-limited setting will often rely on smear microscopy, symptoms, and clinical examinations only. Variability in the accuracy of empirical diagnosis—and the attendant risk of false-positive diagnosis—is likely to be enhanced in the highly heterogenous private sector. A study in India found that TB diagnosis was more often reliant on clinical opinion and less on bacteriological confirmation [12].

As countries look to expand screening programs beyond individuals self-reporting to TB clinics (ie, passive case finding), the screening population will likely have a lower prevalence of disease, either at the start, or the prevalence could drop after repeated rounds of screening. Table 1 illustrates how such a drop will also affect the rates of false-positive diagnoses, and why different algorithms may therefore need to be considered in these screening programs as compared to evaluating self-reported individuals. If a standard algorithm of symptom screen, sputum microscopy, and empirical diagnosis (Table 1, algorithm 1) was used, a drop from the current approximate 10% prevalence among patients submitting sputum for TB diagnosis in healthcare settings to a high-risk screening population with 1% prevalence (1000/100000) can result in >70% of all TB diagnoses being made among people who do not have underlying TB disease—that is, nearly 2.5 false-positive TB diagnoses for each true positive.

**Table 1.**
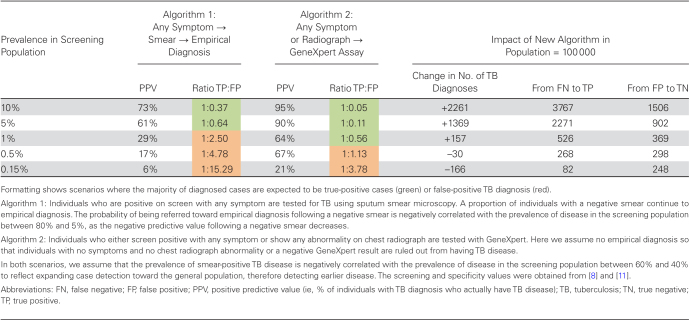
False-positive Tuberculosis Diagnoses in Hypothetical Screening Programs

**Figure 2.**
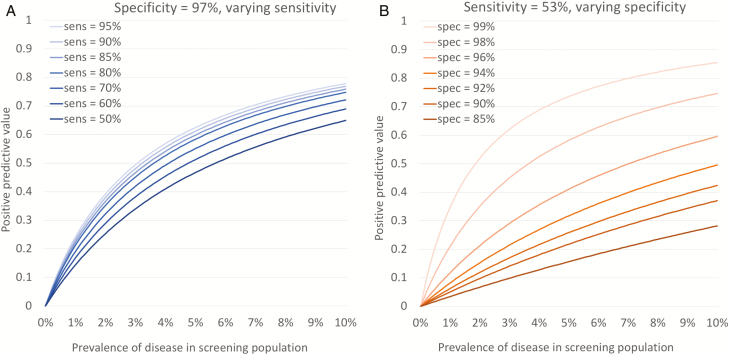
Change in positive predictive value by varying sensitivity (*A*) or specificity (*B*). Figures show relationship between positive predictive value (% of individuals with tuberculosis [TB] diagnosis that actually have TB disease) and prevalence of disease in screening population for combinations of sensitivity and specificity. *A*, Lines show how relationship changes if specificity for algorithm 1 (see Table 1) remains constant at 97% but sensitivity increases. *B*, Lines show how relationship changes if sensitivity remains constant at 53% but specificity increases or decreases.

To mitigate the high probability of a false-positive diagnosis, screening programs could look to increase the specificity, or consider improving sensitivity to diagnose more true-positive cases. Figure 2A and 2B show how the positive predictive value (PPV; ie, the proportion of individuals with TB diagnosis that actually have TB disease) changes with sensitivity (Figure 2A) or specificity (Figure 2B). From these, it becomes clear that improvements in sensitivity will have limited impact on the PPV, and that prevalence in the screening population is the key driver. However, the PPV is much more dependent on small increases or declines across a narrow range in specificity (Figure 2B).

Ideally, diagnostic algorithms in screening programs would improve both sensitivity and specificity, for example, resembling the protocols used in prevalence surveys (Table 1, algorithm 2). However, even with an algorithm of 99.0% specificity, a screening population with a 0.5% prevalence (500/100000) of TB would lead to the number of false-positive TB diagnoses outnumbering true-positive diagnoses.

It is important to note that these scenarios use best available, but sometimes weak, current estimates for the sensitivity and specificity of all tests, including empirical diagnosis [8]. However, in the absence of wide application of improved diagnostic tools, the stronger the external push to increase the number of TB diagnoses (whether by using more sensitive tests or empirically treating more people), the more specificity will become compromised, and the higher the number of false-positive diagnoses will be.

## CONSEQUENCES OF FALSE-POSITIVE TB DIAGNOSIS

### The Individual

False-positive diagnoses for TB are usually considered to have minimal long-term health implications for patients. However, they can lead to substantial negative consequences—consequences that generally will not be later corrected. Specifically, patients testing false positive for active TB will almost invariably be recommended for a treatment course that currently lasts a minimum of 6 months. Not only does TB treatment carry a nonnegligible risk of adverse clinical events (for example, at least a 1 in 50 risk of clinically relevant hepatotoxicity) [13], but patients also incur substantial costs. Even when clinical services are provided to the patient for free (as is the case in many settings), patients still incur high nonclinical costs, including transportation, food, childcare, and lost wages. In a systematic review of patient costs including 14 studies in low-income countries, the mean direct patient cost (plus productivity loss) was estimated at $248 per patient [14]; a second systematic review estimated that total patient costs of TB averaged 58% of annual patient income, with half of those costs occurring after treatment [15]. In addition, undergoing TB treatment (especially when directly observed and in poor structural conditions) often results in damaging social consequences—including stigma, isolation from families, gender discrimination, loss of hope, and disrespect [16].

Less well studied are the consequences of missing other conditions that may be falsely diagnosed as TB. One small study has suggested an increase in mortality among HIV-infected Ugandan adults with false-positive TB microscopy results [17], though this result was not statistically significant. Underlying conditions in individuals falsely diagnosed as having TB can range from bacterial pneumonia to lung cancer [18]. In the case of pneumonia—potentially the most common missed diagnosis—rifampin has some activity against the most common bacterial pathogens isolated [19], but other antimicrobial regimens are more effective. Many of these “missed” conditions are more rapidly progressive or fatal than tuberculosis itself, meaning that in many such cases, the consequences of delayed diagnosis resulting from a false-positive TB diagnosis will be equally (if not more) clinically devastating than those of false-negative TB diagnoses.

In contrast to perceptions around false-positive diagnoses, the consequences of false-negative diagnoses of TB are often portrayed as being fatal. As a result, studies of relative harms often suggest that a large number of false-positive diagnoses should be tolerated to avert a single false-negative diagnosis (with ratios as high as 30:1) [20]. However, indirect evidence suggests that most individuals with TB initially diagnosed as false-negative often later start treatment—either empirically or through other diagnostic tests. For example, results from clinical trials suggest that the advantages of using more sensitive diagnostic testing with the Xpert MTB/RIF assay relative to sputum smear microscopy is at least partially counterbalanced by existing practices of empirical treatment (ie, treatment of patients testing false negative by Xpert) [21–24].

Thus, while much of the TB literature has focused on the potential consequences to patients of false-negative diagnoses, the implications of false-positive diagnoses—from life-threatening side effects to social stigmatization to morbidity and mortality from other conditions—may be no less dire. The relative harm of false-positive vs false-negative diagnosis is an important consideration in, for example, decisions to screen for TB in lower-risk populations or to use more sensitive, less specific tests (eg, Xpert Ultra) [25].

### Household

Households of individuals who receive false-positive diagnoses are also negatively affected. Prolonged treatment for TB leads not only to additional healthcare-related expenditures but also to loss of income for the household, which can cause households to experience catastrophic costs [15, 26]. Such a descent into poverty will have a substantial and long-term impact on all household members including education, income, and health [27].

### Health System

Recent modeling analyses have focused on the impact of resource constraints in the health system on the ability to implement ambitious interventions, including constraints on healthcare staff and beds in the multidrug-resistant TB wards [28–30]. In the context of limited drug supply and healthcare workers, treating patients who received false-positive TB diagnoses may substantially worsen care for patients in other segments of the healthcare system.

### Surveillance and Burden Estimation

Program performance will be overestimated in the presence of false-positive diagnosis, as treating patients with a false-positive diagnosis will have no impact on the TB epidemic. As highlighted by Table 1, reported notifications can easily be misleading as an indicator for the success of an expanded screening strategy—an increased number of notifications may simply represent a dramatic increase in the number of false-positive diagnoses.

In addition, those notified and initiating treatment are assumed to have been at risk of dying due to TB. If a substantial proportion of this cohort does not have TB disease (and instead has less serious conditions), observed mortality rates may be artificially low, leading to overestimates of treatment success both on country and global level.

Such unintentional misrepresentations can, when revealed through, for example, prevalence surveys, undermine faith from domestic and international funders of the national TB response.

These issues also affect burden estimation and reporting at the global level. Current TB estimation methods by WHO and Institute for Health Metrics and Evaluation do not consider false-positive diagnoses, and instead assume that all notified cases represent patients with a true-positive diagnosis [31, 32]. As a consequence, the inclusion of false-positive diagnoses artificially increases official incidence estimates and leads to an underestimation of the gap between incident cases and those notified for treatment [7, 32].

## WHAT CAN BE DONE

### Acknowledge the Problem

The first step will be to explicitly include the challenge of false-positive TB diagnosis in TB policy discourse. In these discussions, policy bodies should explicitly consider false-positive TB diagnosis as a challenge that already inflates reported TB notifications, and acknowledge that these distortions may increase further as new ambitious programs are rolled out. In addition, efforts should be made to estimate the proportion of false-positive TB diagnoses in surveillance data as part of country-level reporting to the WHO Global TB Programme and the Global Fund Against AIDS, Tuberculosis and Malaria. As multiple high-level indicators for policy evaluation and advocacy may be strongly affected by false-positive diagnosis, an explicit evaluation of the potential bias introduced in different settings would be sensible.

To inform such and future evaluations, a concrete step could be to generate a quantitative estimate for the sensitivity and specificity of the diagnostic algorithms as applied in each country, and then to use those estimates to inform estimates of false-positive diagnoses (as in Table 1). While heterogeneity in diagnostic test performance and implementation is likely, strengthening the current data through, for example, programmatic reviews and national strategic planning is of high value. Outcomes could include identified opportunities for improvement (eg, in the protocol for empirical diagnosis) and data strengthening, better estimates of the proportion of false-positive TB diagnoses, and more realistic estimates of country program performance.

### Evaluate Trade-offs in Screening Programs

As outlined in the previous section, false-positive TB diagnoses follow from the trade-off between potential positive and negative effects of real-life screening and diagnostic processes. We propose that rather than focusing solely on the positive potential of new screening strategies, new strategies should be evaluated through a comprehensive conceptual framework that also acknowledges the potential negative consequences of false-positive diagnosis.

Here, any change in screening strategies should be evaluated in terms of delivering the correct diagnosis for all individuals evaluated for TB, including those without TB. In other words, in a setting where a more sensitive screening test is being considered, programs could explicitly consider the aim of increasing the number of individuals who receive a true-positive diagnosis instead of a false-negative diagnosis and balance this against the number of individuals who may receive a false-positive rather than a true-negative diagnosis. Where more specific confirmatory testing is being considered, it is important to consider the number of false-positive diagnoses that could be averted, balancing this against the number of true-positive diagnoses that may be missed.

The practical consequences of these choices are shown in Figure 2 and the final 3 columns in Table 1, where the balance between additional true-positive and true-negative diagnoses shifts notably as the prevalence of disease changes, an observation masked by simply counting the number of additional TB diagnoses.

With each policy decision around, for example, diagnostic approach or population screened, the relative health and financial cost for each diagnosis should be considered. Together, these will provide a comprehensive overview of the cost and benefit of a specific change, for example, informing the choice to try and replace empirical diagnosis with microbiological testing.

In this framework, the trade-offs between sensitivity and specificity of the diagnostic algorithm, as well as the disease prevalence in the screening population, become more explicit. Moving beyond a simple comparison of sensitivity and specificity, the potential performance of new tests or case-finding approaches could be simply summarized in the change in the ratio of false positives/true positives across different prevalences of disease in the target population (Table 1). Alternatively, more sophisticated decision curve analyses can incorporate additional heterogeneity and complexity in given settings [25, 33].

While quantifying the consequences of each trade-off more comprehensively is critical, decisions regarding acceptable levels of false-positive diagnoses (relative to the number of true-positive diagnoses) will still need to be made. Such choices are also multidimensional and highly setting-specific, but explicitly considering these trade-offs represents an important first step.

### Research

Work is needed to provide quantitative estimates for the weight a false-positive diagnosis should carry compared to a false negative, or example, by quantifying the costs and quality-adjusted life years lost through incorrect diagnoses for individuals with vs without TB. Such work could examine the acceptable number of false-positive TB diagnoses for one additional true-positive diagnosis, across settings. A number of papers have already highlighted strong discrepancies between perceived and data-based thresholds, as well as variation between the preferences of clinicians, public health officials, and patients [20, 34, 35].

Another key area of research is to improve the empirical data underlying our current estimates of test performance, particularly the accuracy of empirical diagnosis and the specificity of “definitive” diagnostic tests. While reviews [8] and examples of research exist [36], substantial uncertainty remains on the baseline performance of key tests, particularly as implemented in actual field settings. One example is the specificity of sputum smear, which has shown great variability in recent prevalence surveys, with up to 43% of smear-positive cases not confirmed by culture [37]. Again, some of these data will be setting-specific, in particular for empirical diagnosis. But a better understanding of how the performance depends on specific factors (eg, background TB and HIV prevalence, use/availability of radiograph) will help inform estimates. Recent studies have also highlighted the value of considering urine LAM and C-reactive protein as part of TB screening in high-risk HIV-infected populations [38, 39].

### Test Application

False-positive TB diagnoses are not exclusively caused by suboptimal diagnostic accuracy, but are also driven by pre- and postanalytic errors and administrative errors as well as laboratory errors in handling of reagents and/or maintenance of instruments. By strengthening domestic and international laboratory networks, TB diagnostics can be embedded in well-functioning systems of training and quality control to help reduce false-positive TB diagnoses.

## CONCLUSIONS

As the TB community looks to close the diagnostic gap through expanded screening and improved diagnostic tools, it is important to acknowledge false-positive TB diagnoses as part of current reality and consequence of expanded screening efforts. By recognizing and addressing the size as well as consequences of false-positive diagnoses, we can improve clinical outcomes for individuals and focus the limited resources available to “End TB” to achieve stronger population impact.

## Notes

***Acknowledgments.*** We are grateful for the input during initial discussion on these issues from participants of the TB Modelling and Analysis Consortium meeting in Glion (September 2017).

***Financial support.*** This work was supported by the Bill & Melinda Gates Foundation (TB Modelling and Analysis Consortium: award number OPP1084276 to R. M. G. J. H., D. W. D., N. A. M.); the European Research Council Starting Grant (action number 757699 to R. M. G. J. H.); and the National Institutes of Health (grant number R01 AI112438-01 to N. A. M.).

***Potential conflicts of interest.*** S. S. is employed by the Foundation for Innovative New Diagnostics (FIND), a nonprofit foundation whose mission is to find diagnostic solutions to overcome diseases of poverty in low- and middle-income countries (LMICs). It works closely with the private and public sectors and receives funding from some of its industry partners. It has organizational firewalls to protect it against any undue influences in its work or the publication of its findings. All industry partnerships are subject to review by an independent scientific advisory committee or another independent review body, based on due diligence, Target Product Profiles, and public sector requirements. FIND catalyzes product development, leads evaluations, takes positions, and accelerates access to tools identified as serving its mission. It provides indirect support to industry (eg, access to open specimen banks, a clinical trial platform, technical support, expertise, laboratory capacity strengthening in LMICs) to facilitate the development and use of products in these areas. FIND also supports the evaluation of publicly prioritized TB assays and the implementation of WHO-approved (guidance and prequalification) assays using donor grants. To carry out test evaluations, FIND has product evaluation agreements with several private sector companies for TB and other diseases, which strictly define its independence and neutrality vis-a-vis the companies whose products get evaluated and describe roles and responsibilities. All other authors report no potential conflicts. All authors have submitted the ICMJE Form for Disclosure of Potential Conflicts of Interest. Conflicts that the editors consider relevant to the content of the manuscript have been disclosed.
